# A small switch in perspective: Comparing weight loss by nutrient balance versus caloric balance

**DOI:** 10.5114/biolsport.2024.133666

**Published:** 2024-01-30

**Authors:** James E. Clark

**Affiliations:** 1 Scientific Health: Education and Human Performance. Oakley, CA 94561, USA

**Keywords:** Caloric balance, Nutrient balance, Overfatness, Weight loss, Obesity

## Abstract

The establishment of a Caloric balance has been classically discussed as the means to induce weight loss. Recently, the idea of nutrient balance as opposed to Caloric balance has emerged as a better means to induce weight loss. This investigation compared differences in weight loss between a diet based on a nutrient balanced diet compared to a Caloric balance diet. 53 (27M/26F) active overfat individuals (30.7+/- 7.1 years) were randomly (matched for age, gender, training history) assigned within an 8-week intervention to follow either a self-selected diet (control) or a diet based on following a Caloric balance (%Cal/day) or a nutrient balance (g/kg/day) in conjunction with a periodized exercise regimen to determine effectiveness for each diet to induce weight loss. Nutrient balance group had significantly different changes (p < 0.05) in fat-free mass (2.26 (2.02, 2.49) kg versus 0.42 (-0.40, 1.24) kg) and fat mass (-5.96 (-5.34, -6.58) kg versus -4.08 (-3.92, -5.92) kg) relative to the Caloric balance group and was more effective at meeting nutritional requirements for protein (ES = 0.65 (0.48, 0.85)) and lipids (ES = 0.24 (-0.09, 0.98)) than the Caloric balance group. Nutrient balance was subjectively scored as easier to follow and more likely to be self-selected. Using a nutrient balance diet may be more effective at inducing beneficial body compositional changes and shows being a more self-selected dietary method when compared to a Caloric balance diet. Therefore, it may be a better choice for advice when offering treatments to those who are attempting to lose weight or maintain weight loss.

## INTRODUCTION

We have come to understand the relationship of fatness with the onset of non-communicable diseases [1, 2]. Especially for those individuals expressing overfatness (metabolic dysfunction associated with excessive fatness regardless of designation of obesity) [2]. More-over, we understand that metabolic changes associated with changes in nutrition and increased activity tend to reverse many of the health issues associated with overfatness [2–6]. So much so, that over the last half-century the use diet and exercise to prevent many non-communicable diseases (e.g., diabetes and metabolic syndrome, cardiovascular disease, cancer) associated with overfatness and has become cornerstone of therapy for ameliorating issues of overfatness that lead to the non-communicable diseases [1, 6–12].

Unfortunately, while we have previously reported in meta-analysis [8] that there is a lower level of effectiveness in treatment by simply establishing a hypocaloric condition for changing body mass or body composition, the conversation both within and outside of healthcare and fitness continues to focus on concept of the Caloric balance and one’s overall body mass (or more importantly body composition) [6, 11, 13–19]. So much so that weight loss and maintenance have often been discussed based on the idea of the Calorie balance [12–14, 16, 17]. Where the central tenet focuses on the idea that consumption of more Calories than expended in activities throughout the day would lead to weight gain, while the opposite is associated with weight loss.

From this authors point of view, it appears to be that the simplicity of this idea may be the crux for its continued use. Yet, the rationale has several logical flaws that hamper its overall justification for altering or maintaining body mass [11, 12, 14, 20, 21]. The first being the semantic question that should be constantly asked when the discussion is raised: *how much does a Calorie weigh?* Where it must be remembered that Calories are mass-less units of energy (heat required to increase 1 kg water 1-degree Centigrade) and as such Calories cannot contribute to weight. Beyond this rhetorical point, the largest flaw is that the set point generally used for the balance is not universal for all individuals, e.g., 2000 Cal/day, and using such generalities may give a balance that automatically establishes conditions for overconsumption of food and thus weight gain [2, 7, 14, 20–24]. Moreover, the expected energetic expenditure throughout the day is typically established via one of many regression equations that allows for an estimated set point (i.e., basal metabolic rate) that may not accurately establish a true set point for the person [13, 20–25]. Or accurately reflect the estimated energy balance points for inducing weight loss [26]. As most of the regression equations that have been generalized to the whole population were established based on a non-generalized population. Meaning that for women, minorities, or other under-represented populations using many of the established equations may not provide an accurate representation of estimated energetic needs. Lastly, as have been previously stipulated [4, 8, 10], the use of a hypocaloric dietary interventions without exercise have limited effectiveness at inducing long-term weight loss or body compositional changes.

As such, this idea has begun to be called into question. The premise that has been the foundation to calling into question the validity of Caloric balance is not simply because of the flaw in the estimation of energetic demand, but what the balance does not consider; foods consumed are used for other metabolic processes beyond those associated with energetics [7, 21, 22, 25, 27–30]. As such, we must begin to revisit how to advise food consumption for those attempting to lose body mass. Regardless of when foods are being consumed, or the composition of food that is being consumed [31, 32].

From this perspective, the idea of nutrient balance has come to the forefront as a counter to the idea of Caloric balance [7, 18, 22, 28, 31–33]. This idea is not something new, however the application of the nutrient balance in regard to weight loss and maintenance is. Yet, outside of attempting to support an ill-fated hypothesis on obesity or for a nutrient-for-nutrient replacement [34–36], there have not been many investigations that we have seen to date which compared Caloric relative to nutrient balance diets.

Thus, the purpose of this investigation was to examine the relationship of weight loss and subsequent maintenance based on use of Caloric balance versus using Nutrient balance. Where it is hypothesized that use of nutrient balance allows not only for greater weight loss but will allow for a greater beneficial change in body composition (i.e., maintaining fat-free mass) versus the use of Caloric balance when combined with exercise in a weight loss regimen.

## MATERIALS AND METHODS

### Subjects

Potential participants were recruited through advertisement and postings at fitness centers and gyms from November 2019 through January 2020, September 2021 through April 2022, and January 2023 through March 2023. To ensure power of analysis, a N-size calculation was completed prior to enrollment with the indicated population needed for appropriate power being 38 participants. From 250 interested participants, 55 participants provided informed consent to begin the study, and 53 completed all aspects of the study (1 voluntarily withdrew and 1 did not return a completed excel spreadsheet, both from the nutrient balance condition). To be included in the study, a participant had to be an adult between 18 and 65 years old, overfat (classified as BMI > 25 with WHR or % BF being greater than 10% above “normal” for age and gender). All participants provided medical clearance for participating in diet and wellness program from their personal physician prior to beginning their involvement in the study and were at least moderately active (e.g., moderate physical activity or exercise regimen of at least 45-min 3 or more days per week), had no comorbidity (i.e., non-communicable disease) and were not involved in competitive athletics during the time of the study, table 1.

**TABLE 1:** Demographic information and the measures of interest for body composition (Average+/-SD) for participants in study groups.

	Group					
**Participants (F/M)**	53 (27/26)					
**Age (year)**	30.1 +/-7.37					
**Height (cm)**	167.6+/-10.9					
	**Pre**			**Post**		
**Body Mass (kg)**	86.2+/-19.6			82+/-18		
**Fat Mass (kg)**	32.5+/-9.3			27.2+/-7.6		
**% Fat Mass**	0.37+/-0.05			0.33+/-0.04		
**Fat-Free Mass (kg)**	53.8+/-12.1			54.8+/-11/8		
**% Fat-Free Mass**	0.63+/-0.05			0.67+/-0.04		

	**Caloric Balance Group**		**Nutrient Balance Group**		**Control**	
**Participants (F/M)**	18 (8/10)		16 (8/8)		19 (11/8)	
**Age (year)**	30.65+/-7.49		30.75+/-7.46		29.45+/-6.55	
**Height (cm)**	167.34+/-11.3		167.78+/-10.8		167.42+/-13.5	
	Pre	Post	Pre	Post	Pre	Post
**Body Mass (kg)**	87.16+/-19.13	83.51+/-17.63^^^	84.10+/-18.53	80.0 +/-17.0^*$^^	87.38+/-21.88	83.48+/-20.1^^^
**Fat Mass (kg)**	32.56+/-9.37	28.48+/-7.28	31.25+/-8.69	25.29+/-6.76^*$^^	33.54+/-10.22	29.36+/-8.64
**% Fat Mass**	0.37+/-0.05	0.35+/-0.04	0.37+/-0.05	0.31+/-0.04^*^	0.38+/-0.04	0.33+/-0.03
**Fat-Free Mass (kg)**	54.61+/-12.22	55.03+/-11.9	52.85+/-11.71	55.71+/-11.5^^^	53.83+/-12.9	54.12+/-12.56
**% Fat-Free Mass**	0.63+/-0.05	0.65+/-0.04	0.63+/-0.05	0.69+/-0.04^*^	0.62+/-0.04	0.67+/-0.03

* Denotes significant difference to Caloric group (p < 0.05),

$ denotes significantly different from control (p < 0.05),

^ denotes significantly different from pre-test (p < 0.05)

Following enrollment, and prior to pre-intervention testing, participants were randomly match-assigned (based on matching gender, age, training history, familial/social factors of fatness between groups) to either the control (exercise only and self-selected diet, ad libitum nutrient consumption with no restriction or advice on composition) or dietary intervention group (either nutrient or Caloric balance diets). For those assigned to the dietary intervention, participants were further matched-assigned to follow either the nutrient balance diet or the Caloric balance diet. After assignment, each was provided with an electronic food scale (accurate to 0.01 g), an electronic food log (Excel Spreadsheet), and a standardized exercise regimen [4] to follow during the duration of the study.

All aspects of the study followed Helsinki protocol for using human volunteers and were approved by the independent IRB of Scientific Health: Education and Human Performance (SH-HP-2019-3A) prior to beginning the study. All participants were fully and completely informed about the risks and rewards for participation in the study, participants did not receive monetary rewards for participation but did receive fuel cards to offset cost for transportation for evaluations. Each participant acknowledged their agreement to participate in the study and were aware that they could leave the study at any time without penalty or recourse.

### Study Design

Due to ethical constraints, i.e., recruiting individuals attempting to lose weight into a study where they would be encouraged to halt that behavioral change, the study design did utilize control group that was encouraged to eat as was normal prior to enroll to meet desired weight loss, but did not provide specific details for dietary composition. All participants used a standard exercise intervention and followed either their normal diet prior to enrollment or one of the two dietary interventions for 8-weeks.

### Exercise Regimen

All participants followed a standardized periodized exercise regimen for the 8-week study (with 1-week familiarization) based on a portion of exercise protocol that was previously used to induce long-term body compositional changes [4]. The focus of the training within the periodization was improved endurance coupled with reduced fat-mass and involved 5–6 days/week of exercise with 1–2 days/week of active rest or self-selected endurance activity, see table 2 for breakdown.

**TABLE 2:** Summary of exercise protocol providing weekly details for exercise selection and training parameters (set, rep, %1RM and rest intervals) meant to provide exercise stimulus within the 8-week intervention [4].

Wk	Monday	Tuesday	Wednesday	Thursday	Friday	Saturday	Sunday
1	Lift #1	ET 3-K Easy	Lift #2	MI-ET	Lift #1	ET 5-K Moderate	Active Rest
2	Lift #2	Active Rest	MI-ET	Lift #3	Lift #1	MI-ET	Active Rest
3	CRT1	CRT2	MI-ET	CRT2	Active Rest	CRT1	Active Rest
4	CRT1	MI-ET	CRT2	ET 3-K Hard	CRT1	Active Rest	MI-ET
5	Lift #2	Active Rest	Lift #3	MI-ET	Lift #1	Active Rest	ET or Active Rest
6	Lift #1	Lift #2	Active Rest	Lift #3	MI-ET	Lift #1	Active Rest or ET
7	Lift #2	MI-ET	Lift #3	MI-ET	Lift #1	Active Rest	ET or Active Rest
8	CRT1	MI-ET	CRT2	ET 3-K Hard	CRT1	MI-ET	ET or Active Rest

ET Hard indicates steady state effort at > 75% ${\dot{\text{V}}\text{O}}_{2\max}$; ET Moderate indicates steady state effort 65–75% ${\dot{\text{V}}\text{O}}_{2\max}$; ET Easy indicates steady state effort at 40–60% ${\dot{\text{V}}\text{O}}_{2\max}$.

**Table:** 

Lift		

Lift #1 70% 1RM 3 × 8–10, 60–90 sec rest	Lift #2 70% 1RM 3 × 8–10, 60–90 sec rest	Lift #3 40–50% 1RM 4 × 7–9, 60-sec rest
Bench Press	Incline Bench Press	Squat to Squat Jump

Squat	Dead Lift	Plyometric Push

Lunges (Walking or Multidirectional)	Lateral Step-ups	Power Skips

	Lateral Pulldown	Keg Toss w/ Med Ball

Barbarian Rows	Romanian Dead Lifts	Depth Jumps

Push-Press	Calf Press/Toe Raises	Lateral Push-offs

Super Set: Dips and Arm Curls	Shoulder Circuit (Lateral Raise, to Front Raise, to Rear Raise, I’s and Y’s)	Chest Drops w/ Med Ball

		Split-Stance Squats

		Glut-Ham Hyperextension

Glut-Ham Hyperextension		

Chest Flies		

Core Routine	Core Routine	Core Routine

**Table:** 

CRT							

CRT1				CRT2			

Exercise	Set	Rep/Time	Intensity	Exercise	Set	Rep/Time	Intensity
Warm-up	1	5-min	50% ${\dot{\text{V}}\text{O}}_{2\max}$	MI-ET	1	12 min	
Core Routine	1	1 circuit	N/A	Core Routine	1	1 circuit	N/A
Bench Press	1	25 rep	50% 1RM	Machine Squat	2	25 rep	50% 1RM
Lateral Pull-down	1	25 rep	50% 1RM	Seated Row	2	25 rep	50% 1RM
Smith-Machine Squat	1	25 rep	50% 1RM	Military Press-DB	2	25 rep	50% 1RM
Military Press	1	25 rep	50% 1RM	Push-ups	2	25 rep	N/A
Core Routine	1	1 circuit	N/A				
MI-ET	1	6 min		DB Dead lift	2	25 rep	50% 1RM
Leg Press	1	25 rep	50% 1RM	DB Shrugs	2	25 rep	50% 1RM
Shrugs	1	25 rep	50% 1RM	DB Bench Press	2	25 rep	50% 1RM
Row	1	25 rep	50% 1RM	Lateral Pull-down	2	25 rep	50% 1RM
Dumb-bell Chest Flies	1	25 rep	50% 1RM	Core Routine	1	1 circuit	N/A
Core Routine	1	1 circuit	N/A				
MI-ET	1	6 min		1-Leg Leg Press	2	25 rep	50% 1RM
Dumb-bell Shoulder Raises	1	25 rep	50% 1RM	Flies/Reverse Flies	2	25 rep	50% 1RM
Calf-Press	1	25 rep	50% 1RM	Cable Curls	2	25 rep	50% 1RM
Dumb-bell Bicep Curl	1	25 rep	50% 1RM	1-Arm Triceps Pushdown	2	25 rep	50% 1RM
Triceps Cable Extensions	1	25 rep	50% 1RM	Core-Strength Routine	1	2 circuit	N/A
Core-Strength Routine	1	2 circuit	N/A	MI-ET	1	12 min	
MI-ET	1	6 min

**Table:** 

MI-ET set-up and progression:						

Week	1	2	3-thru-4	5	6-thru-7	8
Total time (min)	6	12	18	24	30	36
Total Intervals completed	1	2	3	4	5	6

	Training Intensity (% ${\dot{\text{V}}\text{O}}_{2\max}$) and time per segment					

Segment #	1	2	3	4	5	6
% ${\dot{\text{V}}\text{O}}_{2\max}$	50–60	90–100	50–60	80–90	50–60	70–80
Time (sec)	30	30	60	60	90	90

Each interval is completed by progression from segment #1 to segment #6 without stopping or resting. Repeated intervals are completed by repeating the segment progression for the required number of intervals without stopping between progressions.						

Core Routine: Progress from 1-set @ 30-secs and progress to fatigue and then add additional set working in cycle from first exercise to last exercise with 15-sec rest between

Crunches; Pelvic Lifts; Planks; Superman’s/Hyperextensions; Jack-knives; Medicine Ball Crunches; Retro-twits.

To summarize the exercise, focused on resistance training using both standard set, rep, rest intervals along with circuit resistance training and a concurrent endurance training using a combination of “steady state” effort and interval endurance exercise.

Each exercise session completed, along with records for activities of daily living (including total steps per day), over the length of the study was logged with a trainer at the gym/fitness center following completion of the exercise session, with a 95.3% compliance rate being seen for the participants (range of 91–100%). To ensure control variability of the exercise component, a comparison of activities of daily living log was done between the familiarization week and the weeks of intervention. Comparison indicated minimal changes (ICC = 0.915) between the familiarization week and the 8-weeks of intervention with reduction in non-exercise related activity accounting for points of disagreement.

### Diet Interventions

Following enrollment and during the familiarization week, participants were randomly matched-assigned based demographic information collected upon enrollment (i.e., age, gender, training history and body mass) to 1 of 2 dietary interventions. Participants were assigned to either follow a nutrient balanced diet or a Calorie balanced diet, or to a self-selected control dietary intervention where advice on nutrient ranges were not provided the participants.

Those assigned to the nutrient balance diet followed a diet pattern based on total amounts of food that should be consumed based on the following breakdown [7, 9, 22, 32, 33, 37, 38]:

– Net Carbohydrate: 2–2.5 g/kg body mass (or 120 g/day, whichever is greater)
– Protein: 1.8–2.2 g/kg body mass
– Fat: 1.0 g/kg body mass (with at least 2 g/day ALA, 0.5 g/day EPA, 0.5 g/day DHA, and 12–17 g/day Omega-6)

Where values for macronutrients consumed were modified over the length of the study based on changes in self-recorded body mass. Additionally, participants were educated for determining Net Carbohydrates, i.e., Carbohydrate load, through subtraction of amount of dietary fiber consumed from the total carbohydrates consumed.

Those assigned to the Caloric balance diet first had their basal metabolic rate (BMR) estimated via Harris-Benedict equation:

– Female: ((9.6*BM + 1.8*H – 4.7*A) + 665) *ACF;
– Male: ((13.7*BM + 5.6*H – 6.8*A) + 66) *ACF.

Where BM = Body Mass (kg), H = Height (cm), A = Age (years, to closest year), ACF = activity conversion factor (energy expenditure beyond resting metabolism, established to be 1.6 for purposes of the study here).

The equation was used to allow for modification to occur to Caloric balance over the length of study without needing direct determination of metabolic rate via gas exchange analysis. Since the intention was to induce weight loss over the 8-week intervention, the BMR value was reduced to 95% of BMR in accordance with previously established parameter [4]. Following determination of BMR, assignment of food to consume was provided to each participant based on the compositional breakdown of [4, 7, 32]:

– 40–60% from carbohydrates
– 20–30% from lipids (with subdivided into 5–10% polyunsaturated, 15–20% monounsaturated, < 7% saturated)
– 10–20% from protein.

Regardless of dietary group, all participants were asked to track consumption of fiber (ensuring at least 30 gram/day) and meeting all recommendations for vitamin and electrolytes consumed (recommended allotments were provided via informational handout).

Each volunteer was provided an electronic food log (Excel worksheet) that allowed for pre-filled cells with expected amounts of food (based on experimental design and equations) to be consumed based either on energetic breakdown (i.e., % of Cal/day) or nutrient balance (i.e., g/kg), or left blank for the control group. Entry logs allowed for documentation of macronutrient consumed and indication for types of macronutrients consumed (e.g., type of carbohydrate or lipid/fat, amino acid compositions) provided by food label documentation. Participants were instructed to log their individual meal intake into the worksheet and were reminded (by a text and email massage, or the trainer following exercise) every other day to log their food to allow for reduced recall bias [39, 40] and ensure reliability and validity of food recall.

A 3-question Likert-scale (strongly agree to strongly disagree) survey was administered every week through email communication to assess: 1) ease to follow recommendations(statement: I found the dietary guidelines easy to follow, regardless of where I have my meals), 2) willingness to self-select to continue diet (statement: I would openly follow this diet after the study has concluded), and 3) ability to understand recommendations (statement: I found the guidelines provided are clear and easy to understand to build my meals throughout the day). The Likert survey was administered to address secondary questions, regarding the subjective idea of following dietary recommendations, along with to serve as a reminder for participants to continue recording their foods consumption.

At the end of the 8-week study, all electronic food logs where collected, and any personal identification was removed for analysis of balance. Given that we have already shown acute (i.e. day-to-day) oscillations in energy balance (and by extension nutrient balance) seem to have limited impact on long-term body compositional and health status [41], the dietary logs were tabulated for weekly averages for each participant for their respective Caloric and nutrient balance. Determination of differences between expected consumption and actual consumption was then performed for these weekly averages for both absolute and percent differences based on the following equation:

Absolute Difference = Average Consumed-Expected

Consumed Percent Difference = (Average Consumed-Expected Consumed)/Expected Consumed.

Where a positive value was an indication of surplus, while a negative value indicated a deficit.

### Body Mass and Compositional Analysis

All participants were asked to report to their initial and post-training meeting in a hydrated state (having consumed 1–1.5 liters of fluid/day +1 liter of fluid for each hour of exercise) for determining body composition. Height, to nearest ¼ cm, and weight, to nearest ¼ g, were measured via standard medical scale and standiometer with subjects in gym clothing and socks (no shoes). All measurements were made after calibration of the scale and were assessed at the same time of day for that individual. Prior to height and weight measurements, participants voided bowels and bladder; during which time a urine sample was collected and used to assess hydration via USG hydrometer, USG (1.004–1.015) to ensure proper hydration status prior to assessment of body composition measurements via bioelectrical impedance (BIA). BIA was measured three consecutive tests via full body impedance (Omron HBF-514C, Omron Healthcare Inc. Hoffman Estates, IL.), r = 0.993[42], using the average of three measurements for recording and analysis. Participants were then advised to re-weigh themselves using a scale of convenience for determining body mass to use for calculation of BMR or determination of nutrients g/kg body mass.

### Data Analysis

Data was tabulated via electronic worksheet for analysis (available upon request and release from participant for external use). All dietary values were converted for comparison based on conversion of Caloric content of food consumed for comparison of energy balance for both groups Additionally, food logs for both groups were tabulated to allow for determining nutrient balance for both groups. With weeks 1 and 8 and averages over the 8-weeks were used for analysis of dietary metrics for both groups. Both pre- and post-study values of body composition and body mass were tabulated for absolute and relative percentage change. The converted values for Caloric content and for nutrient composition, the determination of net positive or negative balance for both Caloric content and nutrient compositions along with body composition measures were imported into R (R Foundation, https://www.r-project.org/) for statistical analysis and measures of central tendency. Differences relative to the control group and then between and within groups for relative balances for Caloric balance and nutrient balance, and for changes in body mass and composition that occurred over the length of the study were analyzed via ANOVA (within groups via repeated measures and mixed (time*group) for between group), p < 0.05. Changes in body composition where analyzed for via Pearson correlation to determine the relationship between changes in composition and the average relative nutrient or Caloric balances (p < 0.05). To address if there were effectiveness of nutrient balance relative to Caloric balance for inducing relative or absolute changes in body composition values (i.e., body mass, fat-free mass, fat mass) a treatment effect and 95% confidence intervals (CI_.95_) was calculated based on the following equation:

Absolute Chang: (μ _nutrient_ – μ _Caloric_)/σ _nutrient_

Relative Change: (μ _Δ nutrient_ – μ _Δ Caloric_)/σ _Δ nutrient_

Lastly, to determine evaluation of appeasement for following dietary intervention a 3 × 2 Chi-square analysis of Likert categorizations (agree/strongly agree; neutral; disagree/strongly disagree) between the two groups was performed (p < 0.05).

## RESULTS

As seen in table 3, there were differences seen between the two test conditions based on food intake for individuals. For all groups, there were no differences in the estimated Caloric requirements, p = 0.8, but there were significance differences in the consumed energy content of foods between the two groups, p<0.05. Along with significant differences in the consumption of carbohydrates (p < 0.01), lipids (p < 0.05) and proteins (p < 0.05) between the nutrient balance and Caloric balance groups. Yet non-significant differences were seen in protein or lipid consumption between the Nutrient balance and the control (self-selected diet) group (p=0.34) at the end of the 8-week intervention.

**TABLE 3:** Relative nutrient (g/kg) and Caloric (% of BMR) balances based on the use of the nutrient (g/kg) or Caloric (Cal/d) balance dietary plans, Average (CI_.95_) for the first and last week of the 8-week dietary intervention.

	Control		Caloric		Nutrient	

	Week 1	Week 8	Week 1	Week 8	Week 1	Week 8
Average Energy Balance-Estimated (Cal/d)	2771.6	2690.2	2792.5	2707.7	2753.1	2681.6
	(2573.8, 2969.5)	(2508.2, 2872.1)	(2579.5, 3005.4)	(2508.2, 2907.3)	(2549.9, 2956.3)	(2493.3, 2869.9)

Average Energy Consumed (Cal/d)	2341.7^*^	2224.1^*^	2772.9	2552	2253.9^*^$^	2154.8^*^$^
	(2128, 2555.5)	(2027.8, 2420.4)	(2512.4, 2937.4)	(2342.9, 2841.4)	(2072.9, 2434.9)	(1988.6, 2321)

Average Carbohydrate Consumed (g/kg)	3.25^*^	2.9^*^	4.42^^^	4.53^^^	2.25^*^$^	2.3^*^$^
	(1.85, 2.7)	(1.8, 2.65)	(4.32, 4.52)	(4.44, 4.62)	(1.85, 2.7)	(1.8, 2.65)

Average Carbohydrate Consumed (Cal/d)	886.92^*^	831.49^*^	1535.9^$^	1489.25^$^	756.9^*^$^	723.62^*^$^
	(817.8, 959.2)	(696.1, 887.8)	(1418.7, 1652.9)	(1379.5, 1599)	(696.1, 817.7)	(667.8, 779.4)

Average Protein Consumed (g/kg)	1.63	1.95^*^	1.61	1.65^$^	2.2*^$	2.3^*^^
	(1.57, 1.84)	(1.51, 2.48)	(1.57, 1.64)	(1.61, 1.68)	(1.4, 2.65)	(2.0, 2.7)

Average Protein Consumed (Cal/d)	562.5	727.6^*^	558.5	541.55	740.1^*$^	727.6^*$^
	(515.9, 608.1)	(652.9, 762.1)	(515.9, 601.1)	(501.6, 581.5)	(680.7, 799.5)	(652.9, 762.1)

Average Lipid Consumed (g/kg)	0.94^^^	1.06^*^	0.89^^^	0.91^^^	1.02^*$^	1.04^*^
	(0.88, 0.96)	(0.79, 1.23)	(0.87, 0.89)	(0.90, 0.93)	(0.7, 1.1)	(0.7, 1.11)

Average Lipid Consumed (Cal/d)	686.9	728.6	698.1	676.9	756.9	723.6
	(637.46, 755.41)	(679.8, 799.4)	(644.9, 751.4)	(627.46, 726.41)	(696.1, 817.7)	(667.8, 779.4)

* Denotes significant difference to Caloric group (p < 0.05),

$ denotes intake significantly different from control (p < 0.05),

^ denotes intake significantly different from expected (p < 0.05).

**FIG. 1:**
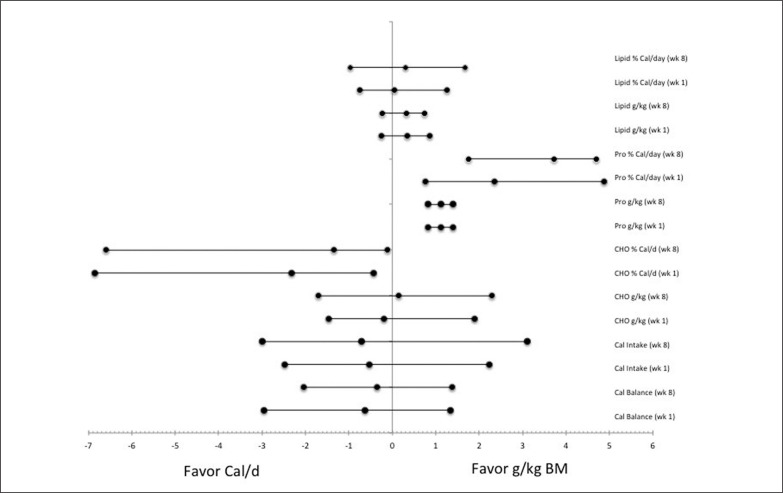
The favorability for effectiveness in following the nutrient balance versus Caloric balance diet has on meeting energy (% Cal/d) and nutrient consumption (g/kg) from carbohydrates (CHO), proteins (Pro), and lipids requirements during week 1 and week 8 of the 8-week dietary intervention.

**FIG. 2:**
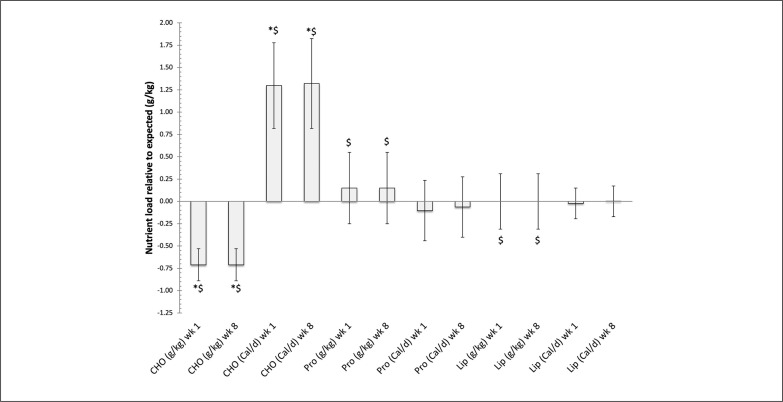
The relative difference in nutrient load in diet versus expected load (Average+/-SD) while following either a Caloric balance (Cal/d) or nutrient balance (g/kg) diet during week 1 and week 8 of the 8-week dietary intervention. Note: CHO = carbohydrates, Pro = Protein, Lip = Lipids * Denotes control group significantly different from Caloric balance group, p < 0.05 ^$^ Denotes nutrient balance group significantly different from Caloric balance group, p < 0.05.

The Caloric balance diet significantly overconsumed carbohydrates relative to their required nutrient load (p < 0.03) and was closer to what would be expected in an athletic individual not attempting to lose body mass, which was also significantly greater relative to the nutrient balance cohort at week 1 and week 8, table 4, ES of 4.43 (3.3, 5.6) and 4.6 (3.42, 5.81), respectively. Along with the Caloric balance cohort consuming 0.09 g/day less than the expected amount of lipids (1.0 g/kg per day) in the diet. Even though it was within guidelines for g/kg per day, the Caloric balance cohort also consumed significantly less protein per day (p < 0.05) than was consumed by the nutrient balance cohort at week 1 and week 8, table 3 and figures 1–2, ES of 4.5 (3.34, 5.67) and 4.68 (3.47, 5.9), respectively.

Nutrient balance diet showed a significant difference in total Caloric intake relative to estimated BMR values, average (CI_.95_) for week 1 -499.2 Cal/d (-558.8, -439.6) and week 8 -526.9 Cal/d (-587.7, -466.1), (p < 0.01). This difference also meant that nutrient balance was significantly more effective intervention to reduce Caloric intake relative to the Caloric balance diet (p < 0.01), ES of 4.54 (2.88, 6.24) and 4.73 (3.0, 6.5) for weeks 1 and 8, respectively.

**FIG. 3:**
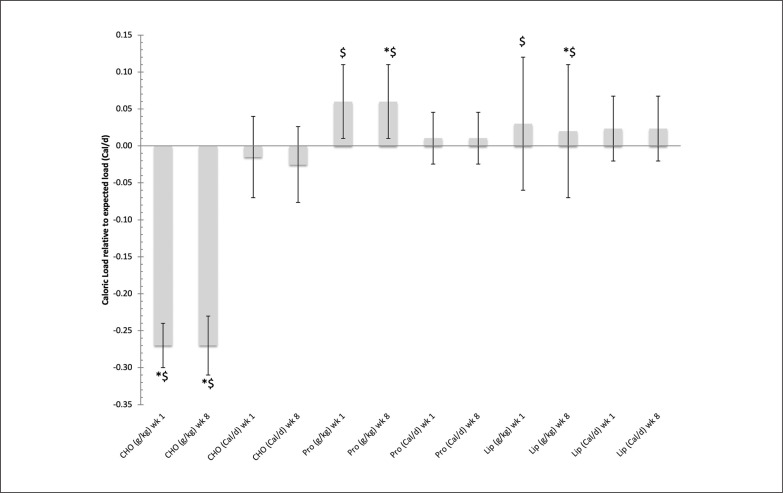
The relative difference in Caloric load in diet versus expected load (Average+/-SD) while following either a Caloric balance (Cal/d) or nutrient balance (g/kg) diet during week 1 and week 8 of the 8-week dietary intervention. Note: CHO = carbohydrates, Pro = Protein, Lip = Lipids * Denotes control group significantly different from Caloric balance group, p < 0.05 ^$^ Denotes nutrient balance group significantly different from Caloric balance group, p < 0.05.

Nutrient balance was able to provide participants with a more effective means meeting the nutrient demands for the individual volunteer, figure 2. While at the same time the use of Caloric balance negatively impacted the ability for the participants to meet their nutrient demands, figure 3. This was in particularly seen with meeting protein requirements for the %Cal/d group.

Additionally, see table 3 and 4, the group that followed the nutrient balance showed a significant difference in the change in the relative values of body composition (i.e., percent fat mass, fat-free mass) relative to the Caloric balance group, p < 0.01. That continued for absolute changes in fat-free mass, p < 0.01, and trends toward being significant for fat mass, p = 0.10.

Nutrient balance diet was shown to be more effective for retaining fat-free mass both absolute and relative percentage, ES of 2.85 (2.11, 3.58) and 0.67 (0.50, 0.84), and reducing the relative fat mass, ES of 0.56 (0.40, 0.70), table 3. Yet, since the CI.95 crosses 0.00, there was no indication for greater effectiveness to induce a change in body mass, ES of 0.23 (-0.23, 0.70).

**TABLE 4:** Changes in the body compositional measure of interest, Average (CI_.95_), based on the nutrient and Caloric balances testing conditions for each group.

Measures of Body Composition	Caloric Balance Group	Nutrient Balance Group	Control Group
Absolute Change Body Mass (kg)	-3.65 (-4.92, -3.22)^$^	-4.1 (-4.77, -2.63)^^$^	-3.39 (-5.38, -1.39)
% Change Body Mass	-0.05 (-0.04, -0.06)	-0.04 (-0.03, -0.05)	-0.05 (-0.06, -0.04)
Absolute Change Fat Mass (kg)	-4.08 (-3.92, -5.92)	-5.96 (-5.34, -6.58)^*$^	-4.18 (-4.06, -5.09)
% Change Fat Mass	-0.06 (-0.05, -0.06)	-0.07 (-0.06, -0.08)^^^	-0.05 (-0.06, -0.05)
Absolute Change Fat-Free Mass (kg)	0.42 (-0.40, 1.24) ^$^	2.26 (2.02, 2.49)^*$^	0.29 (0.28, 0.30)
% Change Fat-Free Mass	0.01 (0.00, 0.01)	0.03 (0.02, 0.04)^*$^	0.00 (-0.01, 0.01)

* Denotes significant difference to Caloric group (p < 0.05),

^ denotes trending toward significant difference to Caloric Balance (p < 0.10),

$ Denotes significant difference relative to control (p < 0.05).

There were non-significant correlations for the change in body mass for both absolute and relative differences (percent change) to the difference in Caloric balance or for the nutrient balance of each macromolecule. There were only two significant, but moderate, correlations noted for body composition values (e.g., fat mass, fat-free mass). One occurred between nutrient balance for carbohydrates with changes in fat mass (r = 0.53). The other with protein balance relative to fat-free mass (r = -0.49). Both seen in the nutrient balance group, only.

The subjective nature of the diet also indicated favorability for the nutrient balance versus the Caloric balance for understanding the diet (χ^2^ = 13.77, p < 0.02) and the ease to follow (χ^2^ = 8.25, p < 0.05). Yet, there was no difference in the preference to follow a nutrient balance or a Caloric balance diet (χ^2^ = 3.023, p > 0.1).

## DISCUSSION

The use of diet and exercise in concert with each other has become the hallmark for lifestyle intervention related to health issues associated with overfat [1, 2,5, 9, 15, 43–45, 46]. Within these interventions there has tended to be hyperfocus on weight loss as a primary treatment goal. A goal which historically centered on advice for weight loss and maintenance related to the principles of the Caloric balance [12–14, 16, 17]. However, this idea has recently come into question. As we have started to recognize logical flaws in the argument that changes in a mass-less unit (i.e., Calorie) can lead to changes in body mass for an individual [7, 9, 11, 12, 21, 22, 30, 32, 47]. Along with our better understanding that neuroendocrine and regulatory functions have an effect on both body composition (i.e., fat mass, fat-free mass) and total body mass can occur relatively independently of one’s energy balance set-point [9, 22, 29, 32], or how energy set-point comes from flaws in determination or estimation either during a period of weight loss or under conditions of weight maintenance [7,15, 23, 26, 47–49]. Not to mention the inherent risk of using weight and mass as reinforcers for the continuation of lifestyle treatment, as the scale can serve as a negative reinforcer, leading to the termination of beneficial lifestyle choices (i.e., better diet and exercise selections) [50–53]. As such, we suggest that it is time to rethink the advice and recommendations that we provide those seeking weight loss or weight maintenance and determine if advice based on a nutrient balance would be more beneficial than the Caloric balance recommendation in a condition where weight loss was a goal of a diet and exercise intervention.

As would be expected based on the previously reported findings [4, 6, 8, 10–12], there was a general trend for all participants to experience some degree of weight loss over an 8-week diet and exercise intervention. Yet, there is a small non-significant correlation (r = 0.08) between the use of a hypocaloric diet and the change in body mass (e.g., weight loss) or the percentage of mass change (r = 0.21), regardless of the method employed. Meaning that while weight loss occurred, the relationship of the negative Caloric balance with weight loss may be independent to each other, even if others have offered for a possible cause-and-effect relationship [3, 15, 26, 49]. Yet this speculation drawn from the findings here is a position that agrees with previously reported findings and a speculation that we may be addressing the treatment concepts incorrectly when discussing dietary roles for altering body mass or health issues associated with overfatness [4, 8, 48].

When these relationships were parsed into the method of dietary intervention, there were correlations noted for alternations on body composition with specific aspects of the dietary interventions. In which, an interesting relationship becomes evident from the results here. Even if the discussion of intervention was to focus on developing a hypocaloric diet, by focusing on nutrient balance (g/kg body mass) one was able to meet minimal requirements for macronutrients (i.e., carbohydrates, proteins, lipids) [7, 9, 29, 54] more effectively than with a Caloric balance focused diet. As consumption of nutrients based on g/kg body mass allowed one to meet metabolic needs, yet at a significantly lower total Caloric density for foods consumed each day. A phenomenon that occurred even though the focus of dietary advice in both interventions was to ensure a set-point within a recognized balance, as the use of Caloric ranges, e.g., 40–60% of Calories from carbohydrates, 10–20% of Calories from proteins, 20–30% of Calories from lipids proposed by the authors advocating for the Caloric balance [7, 29, 32]. While we are addressing the difference in terms of Caloric density, the absolute and relative difference from BMR should not be seen as a contributing factor based on what has become a recognized imbalance required for changes in body mass or body composition [12, 14], nor does the conversation typically relate with the changes to metabolic rates during dietary restriction [13, 15, 25, 45]. This focus ignores the metabolic contributing factors may be at play for the differences seen, independent of the relative Caloric contribution to an energy balance. Yet with limited empirical evidence, the idea deserves future investigation, as once again the overall idea of Caloric balance may be a misguided ideal for treatment for individuals attempting to reverse issues of overfatness or attempting lose unwanted body mass.

Moreover, when consumption of nutrients centered on percentage of Calories per day there was on average a 1.5-fold overconsumption of carbohydrates beyond requirements and was similar to what would be recommended for athletes not attempting to lose body mass [29, 54]. The higher carbohydrate consumption was accompanied with an average 0.4 g/kg under consumption of protein relative to requirements for Nitrogen and protein balance [22, 33]. Given the similar physiological stress of the exercise regimen used, these dietary differences may provide the metabolic rationale for the differences seen in changes in body composition between the dietary intervention groups. As excess carbohydrate, and in particular fructose, consumption is a known signal for lipogenesis (independent of hormonal signals) at adipose tissues throughout the body [28, 55, 56]. While at the same time consumption of protein (amino acids) have been indicated as key regulator in maintaining fat-free mass during weight loss [9, 22, 33]. A key difference in nutrients is very important when a goal of lifestyle modification is to ameliorate health issues of overfatness, and failure to make this shift may be detrimental to that goal, as reduction of fat mass with retention of fat-free mass has been shown to be necessary for obtaining this goal [2, 4, 6, 8, 10, 57].

These later points appear to be key here. As others have reported, alteration of physical activity coupled with changes in body composition and not necessarily a change of body mass should be the focus of intervention programs for those who are overfat [1, 3, 5, 6, 10, 11, 57–59]. Additionally, as we have previously indicated along with other authors, focusing on non-body mass metrics appear to induce continuous employment of interventions in otherwise classified yo-yo’ers [4, 53]. As using non-body mass metrics for goal-setting may encourage continuous use of diet and exercise that otherwise would have been abandoned without supervisory coercion from an authority figure (i.e., medical or fitness professional) [4, 53, 60–62].

Which is furthered by the idea that when seeking long-term interventions there is the need to produce psychological appeasing plan that would lead to a self-selection for continuation beyond the supervision period [6, 63–66]. As such, if dietary interventions are to be incorporated, they must be not only effective but provide mechanisms that would encourage use outside of the intervention and across multiple environments [50, 53, 60, 67, 68]. Something that we have previously reported as it related to the use of exercise for historical yoyo’ers [4] and was also found here regarding dietary interventions. Subjectively, participants showed a greater likelihood to select a nutrient balance (g/kg) diet versus a Caloric balance diet. Where common rationale given during exit interviews included the ease of not needing to perform calculations to determine Calories contained in foods (as previously performed), ability to learn portion size, and readily prepare meals using portion sizing when following the nutrient balance; while those following the Caloric balance diet offered that the diet plan was onerous in needing to not only weigh food but then compute Calories without knowing if they were obtaining the macronutrients necessary. Moreover, the control group which followed a self-selected dietary plan used dietary intake that mirrored the nutrient balance group’s intervention. Taken together, these points indicate that recommendations following a nutrient balance might intrinsically be more appeasing and thus more likely to be followed over the long-term. A point that is necessary should we seek to have shortterm interventions establish lifestyle changes that can be carried over beyond the end of the intervention. An issue that has received limited investigations that deserves more attention for long-term benefits.

While we have positive results, there are some limitations to our study. Unfortunately, there has been limited investigation to compare our responses with and the investigation should hopefully serve as foundation for future research on the subject. Dietary information came from diet recall logs that have limitations in themselves. Additionally, we had intervention groups where all participants showed intrinsic motivation to have improvements in their body composition and overall health status that might not allow for generalization to lesser-motivated individuals [52, 67, 69, 70]. Additionally, because of the ethical limitations of not recruiting participants actively attempting to lose weight into a study where they would not be encouraged to lose weight, we did not have a traditional control group (as we allowed participants to participate in the exercise regimen but follow a self-selected dietary method without advice on ranges of intake for macronutrients to function as our control here) to compare the two dietary interventions with. Moreover, hormone changes were not evaluated here and given the role of the regulatory processes in both body weight and overall health status it is difficult to stipulate unequivocally that nutrient balance would elicit the most beneficial responses to improve overall health status. As such, it is recommended that future research on this subject occur.

## CONCLUSIONS

When used in conjunction with an exercise regimen following a diet based on nutrient balance (g/kg body mass) is more effective than following a Caloric balance (% Cal/day) for inducing body compositional changes in individuals who are actively attempting to lose weight. Additionally, those that followed the nutrient balanced condition were more likely to meet nutrient requirements for normal metabolic functions that would ensure body compositional changes would maintain fat-free mass and possibly improve health status. The use of nutrient balance as a diet mechanism appears to have a higher degree of self-selection that should allow for maintaining of the dietary modifications outside of the intervention window. Thus, using nutrient balance of g/kg may be a more effective means to allow body compositional changes and fat loss that may ameliorate issues of overfatness and may be a more valid means to recommend when discussing a dietary intervention to lose, and then maintain the new, body mass.

## Data Availability

All methodology for conducting the study is available upon request. Data is available upon request and release of information by study participants for outside analysis.
